# A rare giant geode of humeral head in an uncontrolled rheumatoid arthritis: a case report

**DOI:** 10.1186/s12891-023-06695-1

**Published:** 2023-07-14

**Authors:** Fernanda Junqueira Cesar Pirola, Murillo Dório, Ricardo Fuller, Jayme Fogagnolo Cobra, Lucas Peixoto Sales, Camille Pinto Figueiredo

**Affiliations:** 1 Department of Medical Science, Medical School, Pontifícia Universidade Católica de São Paulo, Sorocaba, Brazil; 2 Instituto de Reumatologia de São Paulo, São Paulo, Brazil; 3 Rheumatology Division, Hospital das Clínicas da Faculdade de Medicina da Universidade de São Paulo, São Paulo, Brazil

**Keywords:** Geode, Bone erosion, Shoulder arthritis, Synovitis, Rheumatoid arthritis

## Abstract

**Introduction:**

Rheumatoid Arthritis (RA) is a chronic inflammatory disease depicted by peripheral bone erosive damage leading to joint destruction, deformity and functional impairment. Shoulder involvement is less frequent than hands, wrists and feet, and relevant joint damage may be underdiagnosed if a lower threshold for careful analysis of this joint is not settled, especially in uncontrolled disease.

**Case Report:**

A 70-year-old male with a difficult-to-manage RA since 2010, presenting severe shoulder arthritis with MRI showing a striking giant geode in the left humeral head.

**Conclusion:**

An impressive MRI image showing a giant geode in poorly controlled RA should alert rheumatologists to raise suspicion of shoulder involvement for early investigation and treatment.

## Background

Rheumatoid arthritis (RA) is a chronic inflammatory disease characterized by arthritis and bone erosions, especially in peripheral joints leading to deformities and impaired physical function [1]. The disease is marked by periods of remission and flares. Even if treatment is defined, it has been demonstrated that bone damage is a continuous process, with synovial membrane and cartilage being the most compromised structures preceding bone erosions [2].

The classic pattern of RA clinical presentation is pain, morning stiffness, and symmetrical arthritis in small joints of the hands, wrists, and feet. Large joints, such as shoulders, are often affected in more advanced stages of RA, or in some oligoarthritic form of the disease [3]. In the shoulder, a complex and non-weight-bearing joint, the identification, and quantification of damage can be a challenge and underdiagnosed. Therefore, imaging tools are crucial for detecting structural damage of this joint [2, 3].

Radiography is commonly used to evaluate joint damage, showing bone erosion, joint space narrowing and periarticular osteopenia in RA [4]. However, magnetic resonance imaging (MRI) and ultrasound (US) are also frequently used for additional evaluation [4]. MRI and US allow an early detection of synovitis and erosions with higher sensitivity compared to x-ray. MRI can additionally identify bone edema, a joint damage predictor, besides cartilage loss and tendon involvement [3].

Geodes are subarticular cystic lesions caused by inflammatory changes in the synovial lining responsible for bone and cartilage destruction. The finding of large geodes in shoulder MRI of RA patients has been only rarely described [3].

## Case presentation

This article will discuss a 70-year-old male diagnosed with rheumatoid arthritis (RA) in 2010 with elevated titers of rheumatoid factor and antibodies against cyclic citrullinated peptides (anti-CCP). The patient’s disease was difficult to control despite prior treatment with corticosteroids and approved doses of hydroxychloroquine for 2 years, methotrexate for 3 years, leflunomide for 7 years, and adalimumab for 6 years. As a result of corticosteroid use, he presented with osteonecrosis of the hip that required total arthroplasty.

**Fig. 1:**
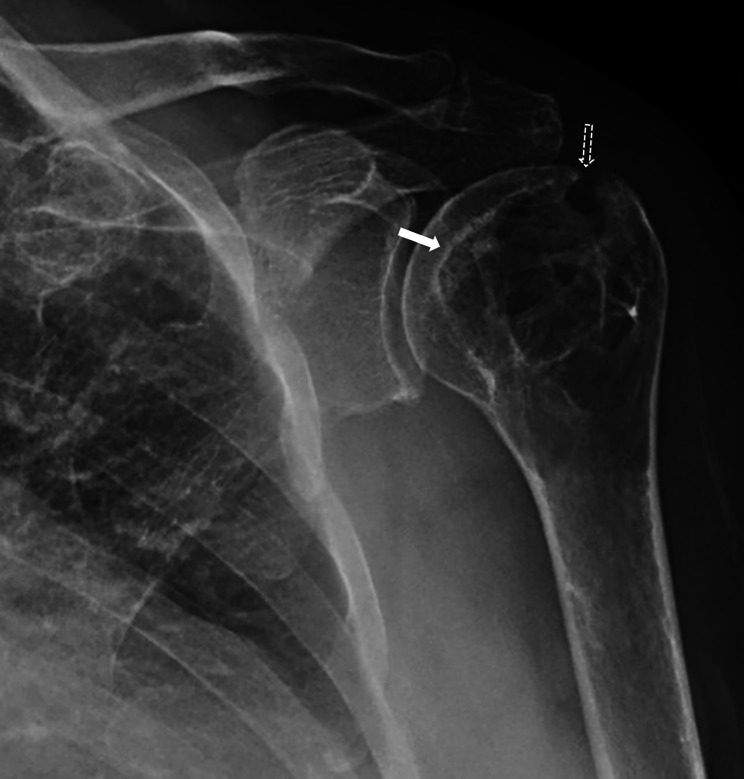
X-ray of the left shoulder in the RA patient. Anteroposterior radiograph shows expansive intramedullary osteolytic lesion, at the metaepiphysis region of the humerus, with well-defined contours and sclerotic border (white arrow), presenting internal septations. The image also shows an erosive lesion on the superolateral region of the humeral head, with slightly cortical thinning alongside (dot arrow)

**Fig. 2:**
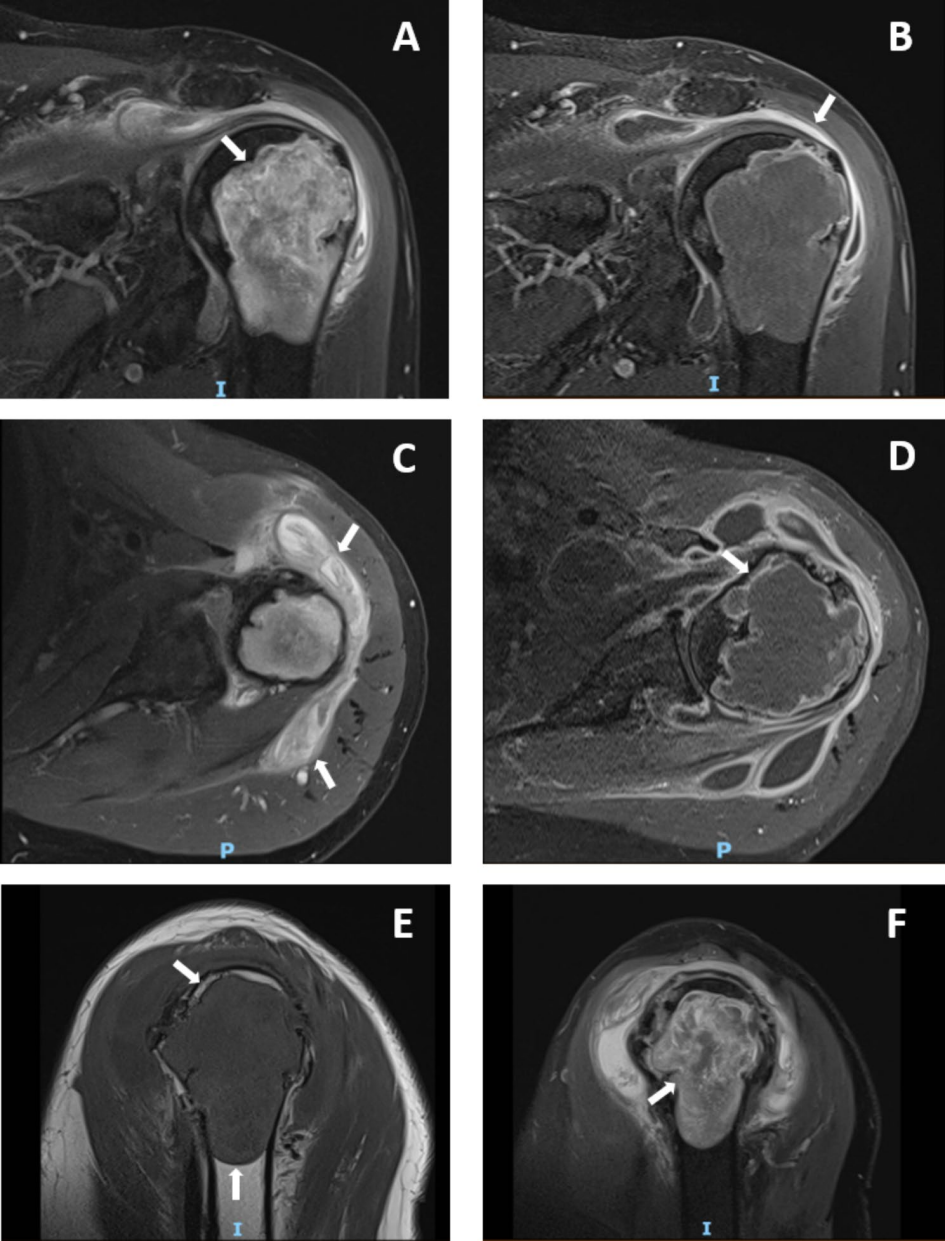
Magnetic resonance imaging (MRI) of the left shoulder in the RA patient. Coronal T2-weighted MR image **(A)** shows a high signal intensity, heterogeneous and well-defined intramedullary bone lesion with sclerotic border (white arrow). Coronal T1 contrast-enhanced **(B)** and Axial T2-weighted MR images **(C)**, both shows a subacromial-subdeltoid bursa distension (white arrows). Axial T1 contrast-enhanced MR image **(D)** shows a high signal intensity, heterogeneous lesion (geode), with peripheral synovium enhancement (white arrows). Sagittal T1 MR image **(E)** show an iso signal intensity, intramedullary lesion, with well-defined contours surrounded by a sclerotic border (white arrow) in the proximal metaepiphysis region of the humerus. Sagittal T2-weighted MR image **(F)** shows a high signal intensity, intramedullary lesion, representative of a geode, fulfilled with synovium (white arrow)

In 2018, he was hospitalized for treatment of an acute interstitial lung disease, which was defined as organizing pneumonia, and his current leflunomide and adalimumab were withheld for approximately 2 months due to possible association with the interstitial lung disease and concomitant infection. To control RA joint disease and interstitial lung disease, the patient maintained only high glucocorticoid doses (methylprednisolone 80 mg/day with progressive dose reduction). In the following months, the patient presented a persistent flare with symmetric polyarthritis in peripheral joints and a severe painful bilateral shoulder arthritis, with high disease activity, as indicated by a Disease Activity Score 28 (DAS28) of 6.42. X-ray of the left shoulder revealed the bone destruction (Fig. 1), and the MRI confirms and provides specific details of the lesion, describing erosions associated with a giant humeral head geode, as well as synovitis, a large bursitis and tendinopathy (Fig. 2).

Sulfasalazine up to 3 gm/day and intravenous abatacept (750 mg/dose) were the chosen treatment. Despite this therapy for 10 months, the patient continued to experience shoulder and hands pain, a high severity activity (DAS28 = 5.45), associated with high levels of inflammatory markers (C-reactive protein [CRP] up to 3.76 mg/dL and Erythrocyte Sedimentation Rate [ESR] up to 106 mm/1 hour), and functional impairment. Abatacept was switched to intravenous Tocilizumab 8 mg/Kg. The patient was also treated with intra and periarticular corticosteroid injection in the left shoulder and intra-articular injection in both wrists, and leflunomide 20 mg/day was reintroduced. In the following months, the patient achieved pain control, normal levels of inflammatory markers (CRP < 0.03 mg/dL and ESR = 3) and disease remission (DAS28 = 1.75).

## Discussion and conclusions

The shoulder involvement in RA patients is less common than hands, feet, and wrists. Herein, the radiological progression with erosive damage and a giant geode, in an aggressive uncontrolled disease, make this case even more unique. The diagnosis was based on MRI findings, that showed in detail both bone and soft tissue damage.

A previous study showed that 12.6% of RA patients developed shoulder arthritis, whether or not associated with other joint arthritis [5]. In addition, symptoms associated with shoulder joint seem to be more commonly found in patients over 60 years old [5]. Another study analyzed the frequency of shoulder erosions in a group of 43 RA patients; the radiographs demonstrated erosions in humeral head in 26 of them and 12 patients had erosions in the glenoid fossa. On the other hand, MRI was able to detect detected erosions in glenohumeral joint in 39 patients and cystic lesions in 15 [6].

Differential diagnoses for cystic lesions of the glenohumeral joint are variable and comprise several conditions. Joint space narrowing, subchondral bone sclerosis, marginal osteophytes, and subchondral radiolucencies are hallmarks of osteoarthritis, while solitary or large cysts are rare. Gout manifests radiographic alterations as a late-stage effect of the disease, with deposits of urate crystals or tophi causing erosions of the underlying bone that resemble geodes or cysts. Intraosseous ganglia are solitary, unilocular or multilocular cystic lesions in the epiphysis of long bones. As a result of joint degeneration, cystic bone lesions may also be present in septic arthritis, synovial chondromatosis, pigmented villonodular synovitis, and amyloidosis. Thus, MRI was of considerable assistance in the reported case [7].

Herein, the patient had a disease control after the association of conventional and an anti-interleukin-6 receptor bDMARD. The last few years have shown that several biological drugs used in the treatment of RA have great impact on controlling disease activity and decreasing joint damage [8]. It has been demonstrated that these agents, known as biological disease-modifying anti-rheumatic drugs (bDMARDs), inhibit damage installation and progression in peripheral small joints, as well as in large joints of lower extremities such as hip and knees [9]. There are no data of bDMARDs specific effects in shoulder joints of RA patients [10].

RA is a chronic erosive inflammatory disease that can cause bone destruction and deformity, predominantly in small peripheral joints. The involvement of large joints, such as shoulders, is less frequent, and it was reported here a rare clinical presentation of a giant geode in the humeral head following an aggressive uncontrolled disease; herein the MRI proved to be a valuable imaging method for identifying damage in both bone and soft tissue, supporting clinical data when other hypotheses may be implicated.

## Data Availability

The authors declare that all data supporting the findings of this study are available within the article.

## List of abbreviations

RA: Rheumatoid Arthritis
MRI: Magnetic resonance imaging
US: Ultrasound
Anti-CCP: Antibodies against cyclic citrullinated peptides
DAS28: Disease Activity Score 28
CRP: C-reactive protein
ESR: Erythrocyte Sedimentation Rate
bDMARDs: biological disease-modifying anti-rheumatic drugs

## References

[1] Tavares Junior WC, Rolim R, Kakehasi AM (2011). Magnetic resonance imaging in rheumatoid arthritis. Rev Bras Reumatol.

[2] Sudoł-Szopińska I, Jans L, Teh J (2017). Reumatoidalne zapalenie stawów w badaniu MR i ultrasonografii. J Ultrason.

[3] Urita A, Endo T, Iwasaki N et al. Giant Geode at the Humeral Head in the rheumatoid shoulder treated with allograft bone grafting and shoulder arthroplasty. J Rhuematol. 2022. 10.3899/jrheum.211082 35428712

[4] Yonemoto Y, Okamura K, Kobayashi T (2017). Predictive factors related to shoulder joint destruction in rheumatoid arthritis patients treated with biologics: a prospective study. Mod Rheumatol.

[5] Ishida K, Nagira K, Hagino H (2021). Rheumatoid arthritis onset from shoulder monoarthritis. Open access Rheumatol Res Rev.

[6] Hermann KA, Backhaus M, Schneider U (2003). Rheumatoid arthritis of the shoulder joint: comparison of conventional radiography, ultrasound, and dynamic contrast-enhanced magnetic resonance imaging. Arthritis Rheum Off J Am Coll Rheumatol.

[7] Bancroft LW, Peterson JJ, Kransdorf MJ. Cysts, geodes, and erosions. Radiol Clin North Am. 2004 Jan;42(1):73–87. 10.1016/S0033-8389(03)00165-9 15049524

[8] Bathon JM, Martin RW, Fleischmann RM (2000). A comparison of etanercept and methotrexate in patients with early rheumatoid arthritis. N Engl J Med.

[9] Lipsky PE, Van der Heijde DMFM, St. Clair EW (2000). Infliximab and methotrexate in the treatment of rheumatoid arthritis. N Engl J Med.

[10] Izumiyama T, Mori Y, Itoi E (2020). Tocilizumab was effective in repairing the large geode in a patient with rheumatoid arthritis. Case Rep Rheumatol.

